# Postsynaptic mechanisms influencing the duration of depolarization discharges in hyperexcitable neuro-glial networks

**DOI:** 10.1186/1471-2202-16-S1-P9

**Published:** 2015-12-18

**Authors:** Vasily Grigorovsky, Berj L Bardakjian

**Affiliations:** 1 Institute of Biomaterials and Biomedical Engineering, University of Toronto, Toronto, Ontario, M5S 3G9, Canada

## 

Hyperexcitability in the neural networks is one of the hallmark features of the epileptic brain and can manifest itself as recurrent short or long duration discharges [1]. In rodent models of Rett syndrome, it was found that long duration discharges were indicative of more severe outcomes which can be used as biomarkers for seizure-like activity [2]. Hyperexcitable networks show an increased expression of potassium afterhyperpolarization (AHP) channels [3] and persistent sodium (NaP) channels [4]. Additionally, astrocytes have been implicated in moderating neuronal firing patterns, exhibiting impaired extracellular potassium clearance in epileptic models and potentially contributing to the development of depolarization discharges. However the precise mechanism that distinguishes depolarization discharges into short and long duration has yet to be fully ascertained.

In this study, we developed a branched computational model of CA3 region of hippocampus, consisting of a network of an astrocyte and a pyramidal cell with a feedback inhibitory interneuron. As both potassium and calcium ions have been shown to potentially affect neuronal hyperexcitability, the astrocytic model has both mechanisms - the clearance of potassium through potassium channels, and the influence of astrocyte in the synapse.

**Figure 1:**
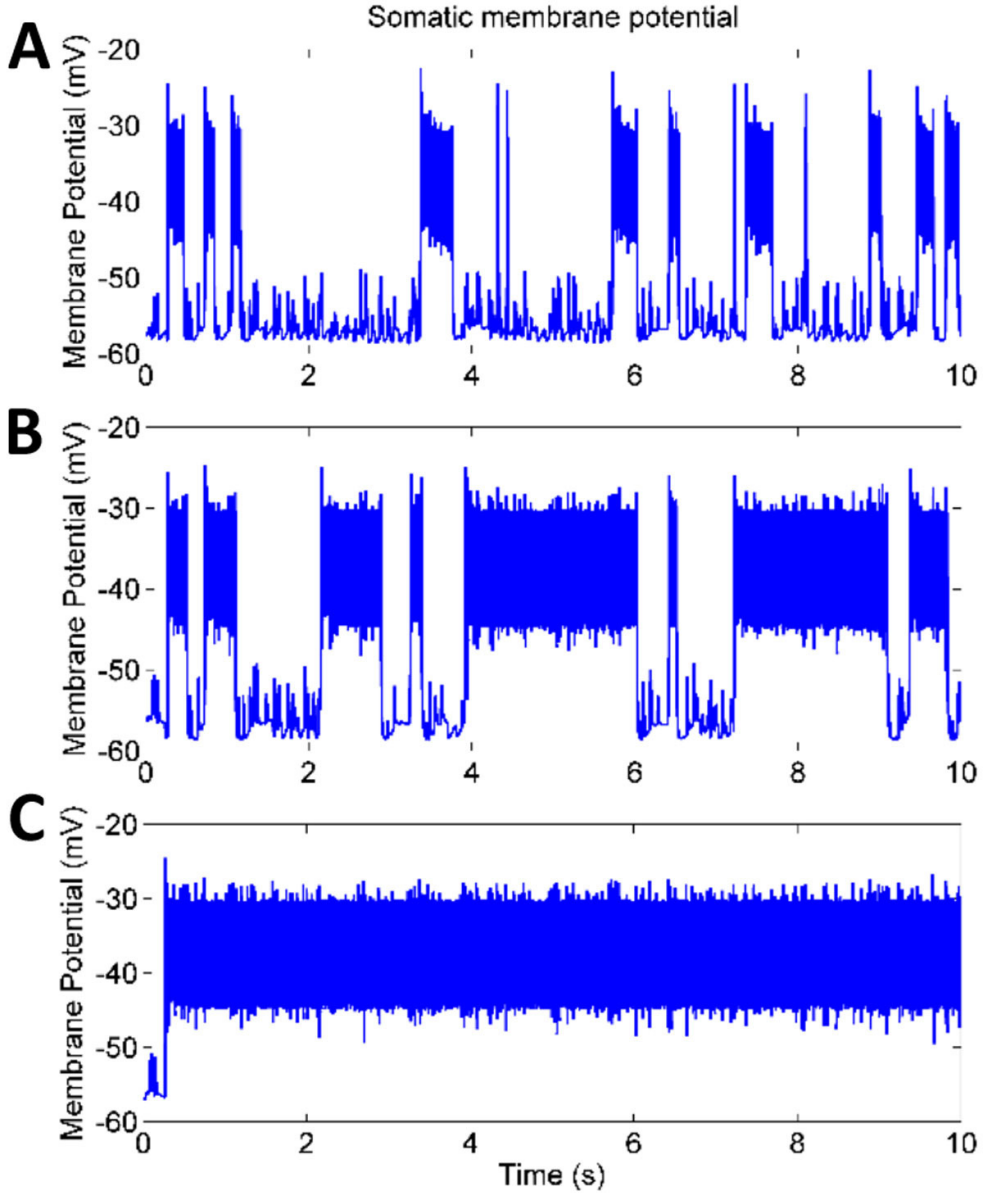
**Effects of AHP channel blockade on the duration of epileptic discharges**. Somatic voltage under A) normal channel expression, B) partial dendritic AHP blockade, and C) full dendritic AHP blockade.

Preliminary results show that in hyperexcitable systems with fully working potassium AHP channels depolarization discharges cease after less than a second (Fig. 1A), classifying them as short duration. With partial dendritic AHP channel blockade the duration of discharges increases, transitioning into long duration discharges (Fig. 1B). In the extreme case where all of the dendritic AHP channels are blocked, there is no cessation of the seizure-like state (Fig. 1C). We hypothesize that in hyperexcitable systems the postsynaptic AHP (and to a lesser extent the Nap) channels work in concert with glial cells to control the duration of depolarization discharges.
